# Current Development and Future Application Prospects of Plants-Derived Polyphenol Bioactive Substance *Curcumin* as a Novel Feed Additive in Livestock and Poultry

**DOI:** 10.3390/ijms231911905

**Published:** 2022-10-07

**Authors:** Shifeng Pan, Jie Yan, Xingyu Xu, Yongfang Chen, Xinyu Chen, Fei Li, Hua Xing

**Affiliations:** 1 College of Veterinary Medicine, Yangzhou University, Yangzhou 225009, China; 2 Jiangsu Co-Innovation Center for Prevention and Control of Important Animal Infectious Diseases and Zoonoses, Yangzhou University, Yangzhou 225009, China; 3 Department of Animal Science, Washington State University, Pullman, WA 99163, USA

**Keywords:** *Curcumin*, biological functions, feed additive, application potential, livestock and poultry

## Abstract

*Curcumin* (CUR) is a kind of natural orange-yellow phenolic compound mainly extracted from the stems and roots of turmeric plants and other species in the genus *Curcuma*, furthermore, it is also the most important active ingredient exerting pharmacological functions in turmeric. In recent years, CUR has been frequently reported and has attracted widespread attention from scholars all over the world due to its numerous biological functions and good application prospects, such as anti-inflammatory, anticancer, antioxidant and providing lipid-lowering effects, etc. In addition, adding a certain dose of CUR to livestock and poultry feed is important for animal growth and development, which plays a key role in animal metabolism, reproduction, immunity and clinical health care. This review aims to summarize, based on the published papers and our own observations, the physical and chemical properties and the biological functions of the plant-derived bioactive ingredient CUR, especially regarding the latest research progress in regulating intestinal health as well as its current development and future application prospects in livestock and poultry as a novel feed additive, so as to provide theoretical and practical references for the further study of the application of CUR as a novel feed additive and a potential new antibiotic substitute, thereby improving the research field of plant-derived bioactive ingredients and promoting the healthy development of livestock and poultry.

## 1. Introduction

The antibiotic resistant is a growing threat which partially results from the harmfulness caused by the misuse or overuse of antibiotics in livestock and poultry production [1,2,3]. China (Announcement No. 194 of the Ministry of Agriculture and Rural Ministry, since 1 January 2020) and many other countries (In the European Union since 2006) have completely banned the addition of antibiotics in the feed production [4,5,6,7], so as to both guarantee safety aspects of all animal-based products, such as meat, dairy, eggs, honey, etc., and maintain public health, and effectively guarantee the healthy development of the modern livestock and poultry industry. However, the prohibition of antibiotics in feed will inevitably cause a series of problems, such as difficulty in controlling related diseases in livestock and poultry production, slow animal growth, decrease the production performance and loss of economic costs, etc., [8,9,10] which speeds up the research and development of harmless feed additives to eliminate or mitigate the influences brought by prohibition of using antibiotics. Therefore, in the era of post-antibiotics, to develop new types of feed additives that are resistant to drug-resistant, non-toxic, non-residual, and find antibiotic alternatives to improve the production performance of livestock and poultry is an inevitable trend of future development [11,12,13].

Numerous studies have confirmed that Curcumin (CUR), the main bioactive ingredients extracted from natural herbal turmeric plants, has important biological functions and future application prospects, and now has become a hot topic for domestic and oversea researchers [14,15,16,17,18]. CUR is a natural polyphenol compound that mainly exists in roots and stems of the ginger perennial plant of the ginger family. It has a variety of biological functions, such as antibacterial, insect-resistant, antioxidant, anti-tumor, anti-inflammatory, blood lipids and glucose lowering effect, enhancing the body’s immunity, improving intestinal health and other pharmacological functions [19,20,21,22,23,24]. It has been reported that CUR and its derivatives play a critical role in inhibiting the mitogen-activated protein kinase (MAPK) signaling pathway and blocking the nuclear factor kappa-B (NF-kB) p65 transferring into nucleus, and thus inhibit the macrophages activation [25]. In addition, CUR can also inhibit bacterial growth and reproduction by destroying the permeability of bacterial cell membranes [26]. Moreover, the addition of CUR in the feed can improve the beneficial effects of probiotics and regulates gut microbiota, predominantly increasing bile acids (BA) metabolism and short-chain fatty acid production associated microbiota, which subsequently increases hepatic cholesterol 7-alpha hydroxylase expression and BA synthesis [27]. Furthermore, CUR was able to significantly reduce cholesterol absorption by downregulating the intestinal Niemann-Pick C1-like protein 1 (NPC1L1) expression, which demonstrating that dietary CUR supplementation shows potential benefits for bile cholesterol supersaturation prevention by regulating gut microbiota and inhibiting intestinal absorption of cholesterol [27]. In addition, CUR is usually used in combination with other drugs or plants derived biomolecules to increase the synergy effects among drugs. A previous study of Hong et al. [28] showed that CUR and lutein nanocapsules combination was able to inhibit the NO production and inflammation reactions in a dose-dependent manner. Therefore, in the context of antibiotics reduction and replacement, the application potential of plant-derived active ingredients as feed additives has become increasingly prominent. However, until now, as reported, literature on the effects and potential application of CUR on livestock and poultry is very limited and is mainly reported in vitro studies, which blocks its rapid promotion as new green additives in livestock and poultry farming. Hence, a solid basis for the assessment of CUR in physiological and productive phases of livestock and poultry in vivo is needed for drawing final conclusions. Therefore, this review systematically summarized the physical and chemical properties as well as biological functions of CUR, especially the latest research progress in regulating intestinal health and future application prospects of CUR as a novel feed additive, so as to provide a theoretical and experimental basis for application in modern livestock and healthy poultry production.

## 2. Methods

In the present review, the preferred reporting items for systematic reviews and meta-analyses (PRISMA) method was adopted for the review which utilized three main journal databases, namely PubMed, Google Scholar and Web of Science. A systematic review has been performed using the above three databases with the descriptors “Curcumin and livestock”, “Curcumin and poultry” and “Curcumin and livestock and poultry”. Inclusion criteria include the article being free full text, published from the year 2012 until 2022 and provided via open access resource or subscribed to by the institution. Retrieved citations were screened, and the data were extracted based on other strict inclusion and exclusion criteria (Table 1). A total of 150 eligible articles were identified for inclusion from the above three databases; after applying the inclusion and exclusion criteria, finally reduced to 90 papers for final inclusion.

## 3. Results

### 3.1. Physical and Chemical Properties of CUR

Turmeric (*Curcuma longa*), also named as “*Curcuma domestica*”, is a herbaceous perennial plant belonging to the ginger family (*Zingiberaceae*) [29]. Although more than 300 active components have existed in this plant, CUR is a substance mainly obtained from the dry roots and stems of the turmeric plant, which has the characteristic of the yellow or orange pigments [30]. Furthermore, it is also the main biologically active compound contributing to medicinal properties and pharmacological functions. It can be divided into autumn turmeric and spring turmeric. In China, it is mainly distributed in autumn turmeric. CUR is actually a kind of orange pigment separated from the roots and stems of the turmeric, accounting for about 77% of the total amount of turmeric pigment (3 to 5% of the weight of the dried turmeric), powder shaped crystals and slightly bitterness [31,32]. It is the most important active ingredients in the turmeric extracts. As a food colorant with the code E100 [33], CUR is commonly used in Asian cuisine, as a main component of curry powder. CUR, a research hotspot in preventing numerous diseases, particularly in cancer treatment, has played vital roles in traditional Chinese and Indian (Ayurveda) medicines for thousands of years (in Asia, usage dates back over 2500 years). CUR has a polyphenol structure and plays a vital role in modulating several biological targets, including certain enzymes, cytokines, growth factors, transcription factors and receptors, protein kinases, and apoptotic proteins, and some favorable results have been obtained from the laboratory studies on the antioxidant, anti-inflammatory and anticancer properties of CUR. CUR could be used as an ideal chemopreventive agent due to its characteristics of being easily accessible, having a low cost and low toxicity. More than 1000 agents have been tested by the U.S. National Cancer Institute for chemopreventive purposes since 1987, and nearly 40 promising agents were finally chosen to clinical trials, among which CUR is one of these agents. However, its poor bioavailability and high potential to interfere with other drugs, as well as the lack of evidence demonstrating its efficacy, constitute barriers in this regard [34,35].

**Figure 1:**
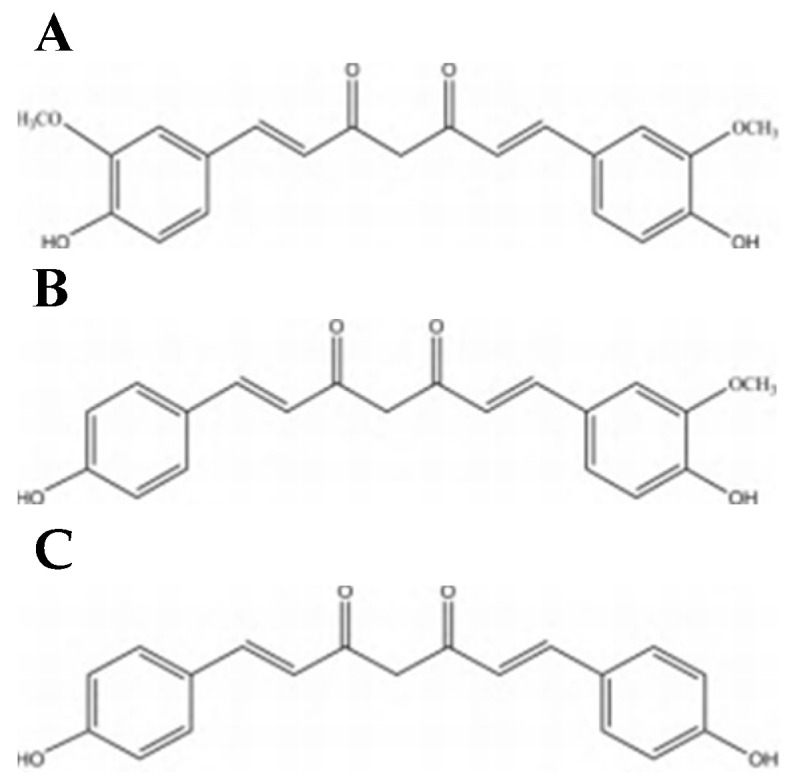
The molecular formulas of CUR and its compounds [40]. (**A**) The chemical structure of CUR. (**B**) The chemical structure of DMC. (**C**) The chemical structure of BMC.

In 1815, CUR was firstly extracted from the rhizomes of *C. longa* by Vogel and Pelletier [32], then in 1842, Vogel Jr firstly purified CUR. Several decades later, in 1910, the chemical structure of CUR as 1,6-heptadiene-3,5-dione-1,7-bis (4-hydroxy-3-methoxyphenyl)-(1E,6E) or diferuloylmethane was reported by Melabedzka et al. In 1913, a synthesizing method for CUR was reported by Lampe and Melobedzka. Forty years later, in 1953, a separation and quantification of CUR components by chromatography was reported by Srinivasan [36]. CUR has two tautomeric forms, the β-diketone and keto-enol, which is practically insoluble in aqueous solutions at neutral and acidic pH at room temperature. In chemical structure, generally mentioned CUR mainly include CUR monomer (C_21_H_20_O_6_), demethoxycurcumin (DMC) monomer (C_20_H_18_O_5_), and bisdemethoxycurcumin (BMC) monomer (C_19_H_16_O_4_) (The molecular formulas are shown in Figure 1). As a kind of lipid-friendly polyphenol compound, CUR is not easy to dissolve in water, but it can be dissolved in alkali, propylene glycol, ethanol and other solutions. Therefore, it can be extracted by organic solvent extraction. Under neutral and acidic conditions, CUR solution is yellow, while in alkaline environments it is brown. Furthermore, CUR is unstable and easy to be decomposed in light, heat, Fe^2+^, etc. The traditional extraction method takes a long time and has a low extraction rate due to the degradation of CUR caused by high temperature. The commonly used modern method is ultrasonic-assisted extraction (UAE) [37]. Firstly, the turmeric rhizomes of the plants were cleaned, dried and were then ground into fine powder and passed through a sieve (20 mesh) [38]. Secondly, the ethanol solution and the powder were mixed at a solid-solvent ratio of 1:25, left standing, extracted using an ultrasonic apparatus. Finally, the extract was obtained by filtration and centrifugation. Shirsath et al. found that the extraction rate was higher when the extraction time was 1 h, the temperature was 35 °C, the ultrasonic power was 250 W, and the ultrasonic frequency was 22 kHz [39].

### 3.2. Biological Functions of CUR

#### 3.2.1. Antioxidation

Factors such as hot and cold stress, feed mildew or pollution, microbial invasion and other factors often induce oxidation stress reactions in livestock and poultry. It is well known that in physiological situations, active oxygen (ROS) and active nitrogen substances (RNS) can be produced by gastrointestinal epithelial cells through oxygen metabolism or intestinal symbiotic bacteria, which play an important role in regulating intestinal health. However, when ROS is over generated, it will cause excess free radicals and cause oxidation stress. A previous study in mice has shown that CUR can enhance mitochondrial antioxidant capabilities by raising mitochondrial membrane potential, reducing mitochondrial dysfunction and apoptosis of liver cells, thereby alleviating acute liver injury [41]. In addition, CUR/TPP-CZL nanomicelles were able to reduce mitochondrial membrane potential significantly, and thus decrease anti-apoptotic protein Bcl2 expression and increase pro-apoptotic protein Bax expression [42]. These above results showed that under different models and treatment concentrations, CUR may play a different regulatory role in mitochondrial membrane potential, and compared with CUR/CZL nanomicelles and CUR alone, CUR/TPP-CZL nanomicelles show better effect, suggesting that CUR/TPP-CZL nanomicelles may become an effective drug delivery targeting mitochondria system of liver cancer cells. In addition, previous research showed that 150 mg/kg CUR adding to the feed can significantly improve the antioxidant performance, egg production performance and egg quality under heat stress in layer chickens. In addition, the results further revealed that CUR-improved antioxidant capabilities were closely related with the reduced serum content of malonic dialdehyde, the increased antioxidant enzyme activity of glutathione peroxidase and superoxide dismutases, as well as increased follicle-stimulating hormone, estradiol, and luteinizing hormone [43,44]. Furthermore, a previous study also indicated that dietary CUR supplementation significantly increased body-weight gain, feed intake, antioxidant enzymes activities and nuclear factor, erythroid 2-like 2 (Nrf2) and heme oxygenase-1 (Hmox1) proteins expression, and then efficiently improved the growth and increased hepatic antioxidant capacity of IUGR weaned piglets [45]. Zhai and others also found that CUR was able to alleviate ochratoxin A (OTA)-induced lipid metabolism disruption and oxidative injury by increasing richness indices (ACE index) and diversity indices (Simpson index) of the cecum and relieving OTA induced decrease in butyric acid producing bacteria abundance, including *Butyricicoccus*, *Blautia* and *Butyricimonas*, thereby restoring the disorders of the composition of the cecum microbiota caused by OTA [46].

#### 3.2.2. Anti-Inflammatory and Pain Relief

The inflammatory process is a defensive response of the body to numerous stimuli. In the last decades, numerous studies have showed that inflammatory cytokines played a key regulatory role in the development of inflammation, among which the tumor necrosis factor-α (TNF-α) and interleukins (ILs) are the most significant contributors [47]. Studies have shown that CUR was able to significantly inhibit the activity of proinflammatory cytokines (including IL-1β, IL-6, and TNF-α), inducible nitric oxide synthase (iNOS) and matrix metal protease (MMPS), which thus significantly inhibited the transcriptional activation of NF-κB signaling pathway and finally inhibited inflammation [48]. In addition, Chen et al. showed that increased number of CD4^+^, CD25^+^, FOXP3^+^ regulatory T cells (Tregs) could be significantly induced by CUR in the spleen tissue of acute lung damage. Further analysis indicated that CUR was able to reduce the inflammatory response during acute lung injury by activating Tregs differentiation and thus adjusting the expression of IL-35 [49]. Another study has shown that dietary CUR supplementation significantly protected the ileum against AFB1 administration induced morphology damage, and decreased plasma AFB1-DNA adducts and eliminated inflammation and oxidation stress in the ileum of ducks. Furthermore, CUR could also be considered as a dietary anti-oxidation and anti-inflammation agent to prevent AFB1 administration induced ileum acute damage, mainly by activating Nrf2-ARE and inhibiting NF-κB pathway [50]. Research on weaning piglets showed that adding CUR to diet significantly decreased the copy numbers of *Escherichia coli*, and the cotreatment of CUR and resveratrol significantly downregulated the toll-like receptor-4 (TLR4) expression in the intestine to inhibit the IL-1β and TNF-α release, and increase immunoglobulin secretion. These results indicated that CUR and resveratrol could ultimately increase intestinal immune function by regulating gut microbiota, downregulating the TLR4 signaling pathway and alleviating intestinal inflammation of weaned piglets [51]. In addition, the anti-inflammatory effect of CUR can also be realized by selectively inhibiting the activity of lipoxygenase, phospholipase A2 and cyclooxygenase-2 [52]. All of the above results consistently demonstrated that CUR could exert anti-inflammatory and analgesic effects in both animal models and clinical trials, and appeared to bring less serious adverse effects than many current analgesics.

#### 3.2.3. Bacterial and Parasitic Infections Inhibition

Bacterial diseases seriously endanger the safety of both humans and animals. Although CUR has no strong and direct killing effect on bacteria, it can significantly inhibit the growth of bacteria, reduce the generation of bacterial virulence factors, inhibit the formation of bacterial biofilms, and prevent bacteria from binding to the host through the bacterial quorum sensing system [53]. Studies have shown that CUR treatments for a long time (24 h) were able to significantly decrease both the activity and the thickness of the bacterial biofilms of *Streptococcus mutans* [54]. Further results showed that long-term use of CUR (24 h) significantly inhibited the formation of bacterial biofilms by downregulating the expression of comC, comD, comE, while the short-time use of CUR (5 min) has no significant effect [55]. In addition, some other studies have shown that the water solubility of the synthetic bifunctional CUR-galactose conjugate is about 11,000 times than that of natural CUR, and compared with traditional CUR, the bifunctional CUR-galactose conjugate has a significantly higher bacterial biofilms suppression rate [56], based on the result that bifunctional CUR-galactose conjugate treatment dose-dependently suppressed the bacterial biofilms formation of pathogenic bacteria, such as *Klebsiella pneumoniae*, *Escherichia coli*, *Pseudomonas aeruginosa*, etc. Furthermore, the minimum bacteriostatic concentration (MIC) of the gram-positive and gram-negative MDR separation strains of bifunctional CUR-galactose conjugate is significantly lower than that of antibacterial drugs such as ciprofloxacin, meropenem, and vancomycin [57]. These above results showed that compared with CUR alone, CUR-semiose puppets have better biological utilization and antibacterial effects.

In vivo results of Eimeria tenella infection showed that with dietary supplementation of CUR alone, or cotreatment with Silan for 37 days and 42 days, the number of coccidia oocyst in chicken feces significantly decreased; furthermore, the survivability of the sporozoites was also significantly reduced. In addition, the CUR-treated chicken showed significantly increased antioxidant level and reduced lipid peroxidation of pectoral muscle, indicating that CUR has a good protective effect on cosmos infection and oxidation damage in chicken [44,58]. In addition, further research showed that CUR has good antibacterial effects, which may be closely related to the reduced aggregation of FtsZ raw wire and the interrupted cell division, thereby inhibiting the proliferation of bacteria. Moreover, CUR can also induce bacterial apoptosis to express a sterilization effect [59,60]. A previous study demonstrated that CUR could increase the apoptosis protein RECA expression in *E. coli*, leading to ROS accumulation, membrane exfoliating and calcium ions, and DNA fragments, and eventually induced the bacterial apoptosis [61]. Further research showed that *E. coli* resulted in DNA damage by recA-lexA-mediated pathway induced apoptosis-like death (ALD), which eventually led to bacterial programming, which indicated that recA-lexA is a DNA damage response coordinator [62].

#### 3.2.4. Lipid Metabolism Regulation

Obesity is one of the most widespread metabolic diseases in the world, which is also the main risk of various metabolic diseases including type 2 diabetes, cardiovascular disease and cancer. It is well known that obesity originates from an imbalance between fat synthesis and lipid decomposition that promotes adipose tissue expansion, and fat synthesis can be regulated by a variety of proteins, such as SREBP-1C and peroxisome proliferator-activated receptors (PPARs), among which PPAR-γ is of great importance for numerous biological processes including preadipocytes adipogenic differentiation, glycolipid metabolism and inflammation reactions [63]. CUR has been shown to have a powerful protective effect against obesity and metabolic diseases. A previous study showed that dietary supplementation with 2000 mg/kg CUR to broiler chickens can significantly reduce the body weight, average daily weight gain, the absolute and relative abdominal fat weight, the plasma content of LDLc and TG, and hepatic TG content. Furthermore, FAS and SREBP-1c gene expression were both significantly decreased, while expression of ACC and ACLY were significantly decreased, in addition, PPARα and cCPT-I expression were also significantly increased [64]. These above results demonstrated that CUR was able to reduce abdominal fat deposition through decreasing the hepatic and plasma lipid profile and affecting the lipid metabolism. Studies on high-fat diets (HFD)-induced obese mice found that CUR treatment significantly prevented HFD-induced obesity and decreased the subcutaneous inguinal WAT (iWAT) and visceral epididymal WAT fat mass. Mechanistically, m6A-dependent TNF receptor-associated factor 4 (TRAF4) expression upregulation by AlkB homolog 5 (ALKHB5) and YTH-domain family 1 (YTHDF1), which thus promoted PPAR-γ degradation by the ubiquitin-proteasome system, contributed to CUR-induced obesity prevention [65]. These above results showed that as a new plant-derived feed additive, CUR may be used to prevent and/or treat fat metabolism disorders and its related diseases in livestock and healthy poultry production.

#### 3.2.5. Gastrointestinal Motion Dysfunction Regulation

The intestine is the longest part of the digestive tract, and different intestines are responsible for the different digestive and absorption tasks. The functions of the intestine can be easily affected by factors such as inflammation, stress and gastrointestinal flora disorders [66], which causes intestinal dysfunction and shows clinical symptoms such as constipation and diarrhea. Yao et al. [67] elucidated the effects of CUR on inflammation associated with both constipation and diarrhea, which were established via cold water gavage for 2 weeks or intracolonic acetic acid (4%) instillation, respectively. The results showed that CUR treatment significantly reversed the elevations in IL-1β and TNF-α in rats with diarrhea and constipation by inhibiting NF-κB. Furthermore, CUR significantly reversed the diarrhea induced MLC phosphorylation in the jejunum of rats, and also significantly reduced TNF-α and IL-1β of rats with constipation and significantly ameliorated the related hypermotility and hypomotility in rats with both diarrhea and constipation. As we all know, intestinal epithelial cells are the first physical barrier for the intestine to resist infringement, and its integrity is directly related to intestinal inflammation. A previous study has shown that CUR was able to protect the integrity of intestinal epithelial cells by activating the transcription factor thermal shock factor 1 (HSF1), and increasing the expression of hot shock protein 70 (HSP70) in the intestinal Caco-2 cells [68]. In addition, Yu et al. also found that intragastric administration of 200 mg/kg/day CUR for 10-20 days significantly improved gastric emptying and atropine (ATR) delayed intestinal propulsion rates in mice. Moreover, intragastric administration of 200 mg/kg/day CUR for 15 days also significantly improved mice gastric emptying and nitric oxide precursor L-arginine (L-Arg) delayed intestinal propulsion rates. No significant effect was found on normal gastrointestinal propulsion of mice after intragastric administration of 200 mg/kg/day CUR for 1–20 days. When normal isolated jejunum of mice was incubated with CUR in vitro, the amplitude of the spontaneous contractile waves of jejunum was concentration-dependently reduced [69]. Taken together, these above results suggested that CUR exerted quite different effects on gastrointestinal peristalsis in vivo and in vitro. Intragastric administration of moderate dose of CUR over 10 days is able to alleviate the gastrointestinal disorders, without affecting normal gastrointestinal propulsion.

**Figure 2:**
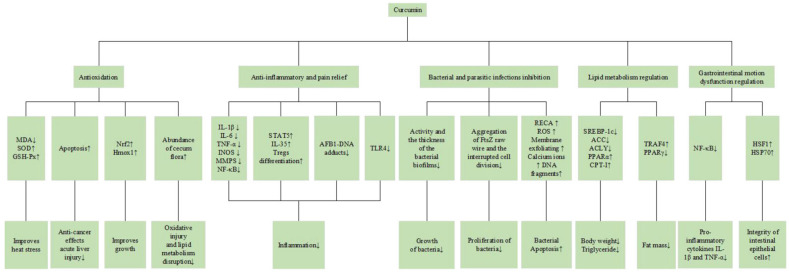
The biological functions of CUR.

In summary, in this section we mainly discuss the biological functions of CUR, based on the nutritional aspects related to antioxidation, anti-inflammatory and pain relief, bacterial and parasitic infections inhibition, lipid metabolism regulation and gastrointestinal motion dysfunction regulation (Figure 2).

### 3.3. Application Research Progress of CUR in the Livestock and Poultry Production

#### 3.3.1. Application of CUR in Poultry Production

Feed additives including chemotherapeutic drugs and antibiotics overuse in broiler diets lead to meat residues, antibiotics resistance complications and other serious side effects. Natural compounds derived from plants could be served as easy and safe substitutes for feed additives. Recent application research of CUR in broiler production is mainly focused on the production performance, immune function, antioxidant and the improvement of meat quality traits. A previous study of Yadav et al. explored the effect of CUR on growth performance, antioxidant status, and gut health of Eimeria species challenged broiler chickens, and found that CUR exhibited some positive responses on antioxidant capacity, lesion score, and oocyst shedding. These above results indicated that CUR was effective in reducing coccidia infection in poultry through its antioxidant and antimicrobial properties, indicating that CUR alone or cotreatment with other feed additives could be recommended as a strategy to improve gut health in broilers [58]. In heat-stressed broilers, Ayman et al. [70] elucidated the impacts of dietary CUR supplementation on energy metabolism, brain monoamines and muscle oxidative stability. The results showed that compared with heat-stressed broilers, increased breast yield and reduced abdominal fat mass were observed in CUR supplemented broilers. Furthermore, the addition of CUR significantly improved monounsaturated fatty acids (MUFAs) and polyunsaturated fatty acids (PUFAs) levels, while decreased malondialdehyde levels in the breast and thigh muscles of broilers, indicating that dietary CUR supplementation could improve carcass yield, energy biomarkers, brain serotonin and muscle oxidative stability of heat-stressed broilers. In addition, Sara et al. showed that 400 mg/kg feed CUR was able to attenuate all the AFB1 modified oxidative stress parameters in the kidney of chicken. Furthermore, high stocking density (HSD) broiler chickens significantly increased the serum corticosterone, malondialdehyde, pro-inflammatory cytokine levels and hepatic leptin expression, while CUR supplementation at a 200 mg/kg diet could significantly improve chickens’ behavioral patterns, growth performance, and immunity by reducing oxidative stress and upregulating the growth-related gene expression of HSD broilers [71]. These above results clearly demonstrated that CUR can protect against stress factors-induced necroptosis and inflammation, and was capable of maintaining the production performance and reducing oxidative stress associated with overcrowding in broilers.

It is known that the metabolic rate of the body is closely related to the blood level of thyroid hormones (T3 and T4). With the reduction in thyroid hormone concentration, the metabolic rate of the body is significantly decreased. In contrast, increasing the level of thyroid hormone can in turn significantly increase the metabolic rate of the body. An interesting report showed that CUR treatment can significantly increase the plasma-free T4 and T3 concentrations in the broiler chicken, increase the metabolic rate and the heat production, thereby reduced the body weight and abdominal fat deposition [64].

In addition, in laying hens, Gong et al. [72] showed that dietary addition with β-carotene, CUR, allicin, and sodium butyrate significantly increased the eggshell strength, egg production, antioxidant activity, immune activity and hormone levels. Further studies have found that the improvement of the quality of eggshell quality by CUR is mainly through the increase in food consumption, which combines calcium with plasma protein or other components in serum, so that there is enough blood Ca^2+^ to form eggshells. These above results showed that dietary CUR can significantly improve egg quality and production performance, as well as the immune status of laying hens. Therefore, CUR can be used as a new type of feed additive to improve the poultry products quality.

Mycotoxin can significantly damage the liver, intestinal tract and the immune function, thereby adversely affecting the healthy breeding of the livestock and poultry industry. Studies have shown that the feed containing mycotoxin ochratoxin A can significantly reduce the content of plasma SOD activity, T-AOC and NO contents in Beijing duck, increase the plasma MDA content, and significantly reduce the height of the jejunum villi, reduce the number of lymphocytes in small intestinal glands, intestinal epithelial cells and the inherent layer, which thus inhibits the iconic immune function and induces intestinal inflammation. However, 400 mg/kg of dietary CUR significantly increased the GSH-PX, T-AOC activity and GSH contents of meat ducks, and reduced the contents of DNA oxidation marker 8-OHdG and lipid peroxide MDA, and thus increased the expression of glutathione transferase (GST) and multiple drug-resistant genes in duck jejunum mucosa, indicating that CUR can significantly reduce the intestinal damage of poultry with mycotoxin. In addition, Jin et al. showed that CUR supplementation is beneficial to the antioxidant capacity of duck meat and growth performance of ducks, dietary CUR raised the duck meat quality, improved the meat color, increased the capacity of water-holding, and inhibited the protein and lipid oxidation, providing new insights into both the qualities and nutrient of ducks of CUR [73]. Therefore, the application of CUR in poultry breeding is not limited to promoting growth and improving product quality, but also effectively reducing the risk of mold toxins in the process of feed production and poultry feeding (Table 2).

#### 3.3.2. Application of CUR in Pig Production

Efficiency and feasibility in the application of plant-derived natural bioactive molecules as feed additives in pig diets has been widely considered in recent years. In the pig breeding industry, the application of CUR is mainly reflected in the regulation of growth and inhibiting virus replication. Studies have shown that dietary CUR supplementation significantly reduced the liver index as well as plasma and liver content of aspartate aminotransferase and lactate dehydrogenase in lipopolysaccharides (LPS)-injected weaning piglets. Furthermore, compared with LPS piglets, total cholesterol and triacylglycerols were decreased by CUR. Mechanically, on one hand, hepatic expression of Bcl-2 and Bax was significantly reduced, whereas the p53 mRNA level was obviously increased; on the other hand, LPS induced enhancement of SREBP-1c and SCD-1 were obviously inhibited. Notably, dietary CUR supplementation significantly decreased FTO, ALKBH5 and YTHDF2 expression, while increased METTL3, METTL14 and the m6A abundance [79], suggesting that the increased m6A RNA methylation contributed to the protective effect of CUR in LPS-induced liver injury and hepatic lipid metabolism disruption. Pig transmissible gastroenteritis virus (TGEV) belongs to coronavirus genus of coronavirus family. Li et al. [80] showed that CUR treatment can inhibit the activity of TGEV virus in dose, temperature, and time-dependent manner. Further results demonstrated the inhibitory effect of CUR on the early replication of TGEV virus and the adsorption of TGEV to the host cells. Swine fever (CSF) is an intense contagious and acute disease in swine, which has the characteristics of high incidence, high mortality rate, and broad popularity and has seriously hindered the healthy development of the pig industry. Even if the vaccine was used widely, the risk of the epidemic and transmission is still difficult to completely block. Its virus infection ability is closely related to the increased expression of the activation transcription factor 6 (ATF6), a key regulator of lipid metabolism, and CUR treatment can significantly inhibit the expression of fatty acid synthesis FASN, and finally reduce the synthesis of ATF6, which suggested that CUR might suppress the duplication of CSF virus by regulating lipids metabolism [81]. Porcine reproductive and respiratory syndrome (PRRS) can cause reproductive failure in sows and severe respiratory diseases in piglets, and is a swine infectious disease that seriously affects economic benefits. Both new piglets and pregnant sows can be seriously infected, which can lead to an increase in the infection rate and mortality rate as well as low survival rate of new piglets [82]. Studies have implicated that CUR can inhibit PRRS virus proliferation in the early days of PRRS through virus internalization and cell fusion. These above results suggested that CUR plays an important role in antiviral and animal health and productivity, which has shown great potential as a natural and sustainable additive in pig diets (Table 3). However, until now, the current research on antiviral effects of CUR on pigs has not yet made breakthrough progress, and from the general trend of CUR research, the effect of using CUR alone is not prominent. Therefore, in the future, preparing the complex of CUR together with other active ingredients can be considered, so as to obtain synergy effects and improve its application potential in pig production.

#### 3.3.3. Application of CUR in the Ruminant Production

It is well known that heat stress seriously affects reproductive performance and animal growth, especially in summer. Jiang et al. [86] explored the effects of dietary CUR addition on the testicular gene expressions and blood biochemical parameters in Hu sheep in summer. The results showed that a basal diet addition with 450 mg/kg CUR significantly increased serum free fatty acid (NEFA), IgA, IgM and IgG content as well as glutathione peroxidase (GPX) level. Furthermore, dietary CUR addition inhibited testicular apoptosis linearly by increasing bcl-2 expression and decreasing caspase-3 expression, which then significantly increased testicular organ index, serum testosterone content, and testicular star expression, indicating that dietary CUR can promote lipid metabolism, antioxidant capacity, immune response and testicular development of Hu sheep, which provides a basis for the application of CUR in sheep production. Another study showed that cur nanocapsules produced by Eudragit L-100 polymer can enhance the anti-inflammatory and antioxidant effects of sheep when used in daily diet, at a dose ten times lower than that of free CUR [87]. These positive effects were reflected in higher total antioxidant capacity and lower lipid peroxidation in milk in sheep-fed CUR-loaded Eudragit L-100 nanocapsules, generating desirable milk properties, which suggested that in practice, the use of nanotechnology enhances the beneficial effects of CUR in milk, possibly creating a nutraceutical food desirable to consumers.

As we all know, LPS is an endotoxin, which may cause immune response and inflammation of bovine mammary glands. Mastitis impairs animal health and results in economic loss. Li et al. showed that CUR significantly rescued the decrease in bovine mammary epithelial cell lines (MAC-T) cell viability and cell damage induced by LPS, further results showed that CUR reduced the accumulation of reactive oxygen species (ROS), the expression of inflammatory cytokines TNF-α, IL-8, IL-6 and IL-1β and the apoptosis rate induced by LPS. These effects were associated with the activation of the nuclear factor E2-related factor 2 (NFE2L2)-antioxidant response element (ARE) pathway coupled with inactivation of the NF-κB inflammatory and caspase/Bcl2 apoptotic pathways. These above results showed that CUR alleviates LPS-induced oxidative stress, inflammation and apoptosis in bovine mammary epithelial cells [88]. Bucak et al. showed that in comparison with control, a 0.5 mM dose of CUR added into bull semen extender led to lower percentage of total abnormality, provided a greater protective effect in the membrane functional integrity and higher levels of the maintenance of total glutathione, while it did not significantly affect the lipid peroxidation and antioxidant potential levels, indicating that antioxidants addition prior to the cryopreservation process might be a recommended strategy to facilitate sperm cryopreservation. Another study has shown that after pretreatment with CUR, sperm movement, vitality, and energy have no significant effects in the frozen semen of the Holshtean bulls, but the ROS content in sperm cytoplasm was significantly reduced, which thus reduced the damage caused by ROS to sperm [89]. These above results deepened our understanding of the biological role and health benefits of CUR and their new potential application in poultry and livestock nutrition (Table 4).

## 4. Conclusions

CUR, a natural polyphenol with numerous biological functions, plays a key role in promoting the growth and healthy development of livestock and poultry, preventing and controlling diseases, and improving the livestock and quality of poultry products. Furthermore, it can also be partially used in antibiotics, antibacterial and insect-resistant drugs, so it has extensive application prospects in the sustainable development of livestock and poultry in the “post-antibiotics” era. Although there are already more detailed reports on the physical, chemical characteristics and biological functions of CUR, most of which are currently limited to cells and animal studies. The application of CUR in the livestock and poultry farming industry needs more animal research to confirm. In addition, the value of its application in actual production still has a lot of room to be developed, and it is urgently necessary to explore the appropriate dosage of various livestock and poultry, the adjustment effect of different livestock and poultry varieties, and its potential mechanisms. Therefore, we need not only focus on the research and development of CUR, which has a high potential for use as new plant-derived feed additive, but also pay attention to the application disadvantages, such as low solubility, difficulty in oral absorption, low biological utilization, and high toxicity that are existent of CUR compounds, and further explore optimization strategies such as nano packaging, emulsification or cotreatment with other drugs, so as to better promote growth and maintain intestinal health functions. In addition, since clinical studies have not confirmed such findings yet, its effect is largely unknown in humans. Therefore, when the favorable results achieved in laboratory studies as well as its advantages including cost, toxicity and availability are confirmed, it would not be wrong to say that CUR is a substance worth being studied. In short, CUR, an essential natural bioactive component with a wide range of biological applications, can be considered as a new type of feed additive and has shown huge application potential, which helps the promotion of livestock and poultry. However, there are also many problems that need to be further solved.

## Figures and Tables

**Table 1:** Inclusion and Exclusion Criteria.

Inclusion Criteria	Exclusion Criteria
(1) Scientific papers published in the form of peer-reviewed scientific articles. (2) Research of experimental and review free full papers can be included. (3) Publications indexed in databases between 2012 and 2022, provided they are in the English language, at least in their title, abstract, and keywords. (4) The articles were searched and summarized by at least 3 people on PubMed, Web of Science and Google Scholar databases. (5) Research on “Curcumin and livestock” and “Curcumin and poultry”.	Documents that are not published in the form of a peer-reviewed scientific article: for example, theses, data briefs, conferences, editorials, opinion articles, etc. The articles were not relevant to the content or had been published more than 10 years should be abandoned. Duplicate items of PubMed, Web of Science and Google Scholar databases should be abandoned. Publications that do not have access to free full text.

**Table 2:** Application effect of CUR in poultry feeding as a plant-derived feed additive.

Experimental Animal	Supplementation Dose	Major Findings	References
Hy-Line brown hens	Dietary supplementation with 100 mg/Kg, 150 mg/kg, 200 mg/kg of CUR	Supplementation with CUR dose-dependently improved egg production by 8.67%, 11.58% and 1.56%, respectively, while the feed conversion ratios decreased by 9.50%, 10.74%, and 2.07%, respectively. Furthermore, the eggshell strength greatly improved by 22.22%, 23.22%, and 26.74%, respectively, and the eggshell thickness improved by 61.49%, 76.40%, and 90.06%, respectively. Antioxidative capability, reproductive hormones and immune parameters, etc. were all significantly increased.	[43]
Broiler chickens	Dietary supplementation with 100 mg/kg, 200 mg/kg of CUR	CUR exhibited some positive responses on antioxidant capacity, lesion score and oocyst shedding, based on the increased growth performance and intestinal permeability, and reduced the lesion scores of duodenum, jejunum and cecum and oocyst shedding. Furthermore, CUR treated chickens had numerically lower oocyst count of *Eimeria maxima*.	[58]
Hy-line brown layers	Dietary supplementation with 250 mg/kg of CUR	Alpha- and beta-diversity of iejunal microbial communities were significantly increased, while *Proteobacteria* and *Bacteroidetes* were significantly decreased. NF-κB in jejunums, and TNF-α in jejunums, the expression of jejunal IL-12 and IL-4 genes were all upregulated. The genes expression of jejunal proteasome activator subunit 3 and 4 (PSME3 and PSME4) was both significantly upregulated.	[72]
Rooster (Ross)	Dietary supplementation with 100 mg/kg of CUR	The dietary CUR supplementation significantly increased the breast yield, but reduced the percentage of abdominal fat. Furthermore, the levels of monounsaturated fatty acids (MUFAs) and polyunsaturated fatty acids (PUFAs) in breast and thigh muscles were both increased. In addition, the dietary CUR supplementation significantly improved the levels of ATP and CoQ10 in liver tissue and brain serotonin.	[70]
Rooster (Ross 308)	Dietary supplementation with 400 mg/kg of CUR	Dietary CUR supplementation was able to almost completely counteract AFB1 induced impairment of SOD, CAT, and GPx. Furthermore, CUR was able to attenuate all the AFB1 modified oxidative stress parameters in the kidney of chicken.	[74]
Broiler chickens	Dietary supplementation with 100 mg/kg, 200 mg/kg of CUR	Growth performance, behavioral patterns, and immunity were enhanced after dietary CUR supplementation by reducing oxidative stress and increasing growth-related gene expression of HSD broilers.	[71]
Broiler chickens (AA)	Dietary supplementation with 300 mg/kg of CUR	CUR significantly decreased the levels of ROS and MDA and increased the activities of SOD, CAT, GSH and ATPase activity, and thus alleviated AFB1-induced liver necrosis by regulating the TLR4/RIPK pathway in broilers.	[75]
Broiler chickens (Cobb)	Dietary supplementation with 200 mg/kg, 400 mg/kg of CUR	Dietary supplementation with nano-CUR significantly attenuated aflatoxin impaired growth performance, blood and serum parameters, carcass traits, and aflatoxin residue in the liver and muscle of broilers.	[76]
Broiler chickens	Dietary supplementation with 1% CUR, 1% acidified CUR	CUR treatment significantly decreased erythrocytes, hematocrit, hemoglobin, ileal coliform and lactic acid bacteria counts, while significantly increased the thymus weight.	[77]
White Pekin ducklings	Dietary supplementation with 200 mg/kg, 400 mg/kg, 800 mg/kg of CUR	CUR treatment significantly prevented the BW and ADG decrease, while decreased the IL-1β, TNF-α and MDA content, and increased the GSH-Px activity in the jejunal mucosa compared with the OTA ducks. Additionally, CUR increased jejunal mucosa occludin and tight junction protein 1 expression, and decreased those of ρ-associated protein kinase 1. Notably, CUR inhibited the increased expression of apoptosis-related genes, and downregulated mitochondrial transcription factors A, B1 and B2 caused by OTA without any effects on RNA polymerase mitochondrial.	[78]

**Table 3:** Application effect of CUR in pig production as a plant-derived feed additive.

Experimental Animal	Supplementation Dose	Major Findings	References
Duroc × Large White × Landrace piglets	Basal diet supplemented with CUR (200 mg/kg diet)	CUR significantly reduced the liver index as well as the plasma and liver concentrations of AST and LDH in LPS injected weaning piglets. Furthermore, CUR attenuated the LPS induced increase in hepatic SREBP-1c and SCD-1 mRNA.	[79]
Duroc × Landrace × Large White	Diet supplemented with CUR (200 mg/kg diet)	CUR significantly decreased the MDA and PC levels in longissimus dorsi muscle improved meat quality and alleviated oxidative stress by activating Nrf2 pathway. Moreover, CUR reduced fat deposition by inhibiting PPAR-γ in IUGR pigs.	[83]
Duroc × Landrace × Yorkshire	Diet supplemented with CUR (200 mg/kg, 300 mg/kg, 400 mg/kg)	CUR decreased feed/gain ratio and crypt depth, improved villus height and crypt depth ratio, reduced plasma D-lactate and DAO activity, increased sIgA expression, increased the number of goblet cells (GCs) and reduced the number of intraepithelial lymphocytes. IL-1β, TLR4 and TNF-α expression were also decreased in CUR pigs, but IL-10 mRNA was increased.	[84]
Duroc × (Landrace × Yorkshire	Diet supplemented with CUR 400 mg/kg	Dietary CUR supplementation increased feed intake, body-weight gain, antioxidant enzymes activities, and the hepatic Nrf2 and Hmox1 expression in weaned piglets with IUGR.	[45]
Duroc boars	CUR in freezing extender (0.125, 0.25, 0.50, 0.75 and 1.0 mmol/L, respectively)	Addition of CUR at 0.25 or 0.50 mmol/L CUR yielded the higher percentage of progressive motility (33.3% and 36.1%, respectively). A significantly higher percentage of acrosome integrity was found in groups administrated with CUR than in the other groups.	[85]

**Table 4:** Application effect of CUR in ruminant farming as a plant-derived feed additive.

Experimental Animal	Supplementation Dose	Major Findings	References
Lacaune sheep	Diet addition 30 mg free CUR/kg concentrate, 3 mg Nano-PCL/kg concentrate, and 3 mg Nano-Eudragit/kg concentrate	The number of total leukocytes and serum globulin levels were lower in 3 mg Nano-Eudragit/kg concentrate than in the control group, antioxidant capacity against peroxyl radicals (ACAP) and catalase enzymes was elevated in 3 mg Nano-Eudragit/kg concentrate, with consequently reduced lipid peroxidation and LPO, and increased ACAP in milk.	[87]
Lacaune lambs	Diet addition ethyl polymethacrylate (Eudragit L-100) nanocapsules loaded with CUR (N-CUR)	N-CUR significantly decreased neutrophil and neutrophil counts, increased serum AST concentrations in lambs. Furthermore, N-CUR obviously decreased the serum blood glucose and triglyceride concentrations, and raised the serum SOD in lamb.	[90]
Hu sheep	Diet addition CUR 450 mg/kg; 900 mg/kg	CUR significantly increased serum NEFA and GPX, as well as IgA and IgM. Furthermore, dietary CUR supplement increased testicular organ index, serum testosterone level, and testicular star mRNA expression. Moreover, dietary CUR supplement linearly inhibited testicular apoptosis with increased testicular bcl-2 mRNA expression and decreased caspase-3 mRNA expression.	[86]
Nili-Ravi buffalo, Angora goats and Holstein bulls	CUR in freezing extender (0.5–10 mM)	At pre-freezing and post-thawing, compared to 0.5 and 1.0 mM CUR and control, 1.5 and 2.0 mM CUR increased total antioxidant contents and decreased lipid peroxidation levels. At post-thawing, rapid velocity and progressive motility were higher with 1.5 mM compared to other doses of CUR. Cryopreservation diluents with antioxidants at three different doses, led to lower percentages of acrosome and total sperm abnormalities, compared to the control. SOD activity was also found to be higher in the presence of CUR at different dose levels and carnitine (5 mM), compared to the other groups.	[85,91]

## Data Availability

Not applicable.

## Abbreviations

ACC, acetyl-CoA carboxylase; ACLY, ATP-citrate lyase; ATF6, activating transcription factor 6; CPTI, carnitine palmitoyltransferase I; CUR, curcumin; FAS, fatty acid synthetase; Hmox1, heme oxygenase 1; HSF1, heat shock factor 1; HSP70, heat shock protein 70; iNOS, inducible nitric oxide synthase; IL-1β, interleukin 1 beta; IL-6, interleukin 6; LPS, lipopolysaccharide; MAPK, mitogen-activated protein kinase; MLC, myosin light chain; MMPs, matrix metallo-proteinases; NF-kB, nuclear factor kappa-B; NPC1L1, niemann-pick C1-like 1; Nrf2, nuclear factor E2 related factor 2; PPAR-γ, peroxisome proliferator-activated receptor-gamma; RNS, reactive nitrogen species; ROS, reactive oxygen species; SREBP-1c, sterol regulatory element-binding protein-1c; STAT5, signal transducer and activator of transcription 5; TG, triglycerides; TLR4, toll-like receptor 4; TNF-α, tumor necrosis factor-alpha; TRAF4, tumor necrosis factor receptor-associated factor 4; Tregs, regulatory T cells; UAE, ultrasonic-assisted extraction.
